# Image Tracking for the High Similarity Drug Tablets Based on Light Intensity Reflective Energy and Artificial Neural Network

**DOI:** 10.1155/2014/304685

**Published:** 2014-07-17

**Authors:** Zhongwei Liang, Liang Zhou, Xiaochu Liu, Xiaogang Wang

**Affiliations:** ^1^School of Mechanical & Electrical Engineering, Guangzhou University, Guangzhou 510006, China; ^2^School of Mathematics & Information Science, Shanghai Lixin University of Commerce, Shanghai 201620, China

## Abstract

It is obvious that tablet image tracking exerts a notable influence on the efficiency and reliability of high-speed drug mass production, and, simultaneously, it also emerges as a big difficult problem and targeted focus during production monitoring in recent years, due to the high similarity shape and random position distribution of those objectives to be searched for. For the purpose of tracking tablets accurately in random distribution, through using surface fitting approach and transitional vector determination, the calibrated surface of light intensity reflective energy can be established, describing the shape topology and topography details of objective tablet. On this basis, the mathematical properties of these established surfaces have been proposed, and thereafter artificial neural network (ANN) has been employed for classifying those moving targeted tablets by recognizing their different surface properties; therefore, the instantaneous coordinate positions of those drug tablets on one image frame can then be determined. By repeating identical pattern recognition on the next image frame, the real-time movements of objective tablet templates were successfully tracked in sequence. This paper provides reliable references and new research ideas for the real-time objective tracking in the case of drug production practices.

## 1. Introduction

Nowadays, the automation level of drug mass production and quality inspection still remains undeveloped; most drug tablets in random position distribution were manually inspected for picking out those unqualified ones and then hand-encapsulated after amount counting or bottle packaging by ocular estimation. All drug tablets were highly similar or identical to each other in shape, size, surface topography, and color, which imposes colossal operation overload and time exhaustion on the working operators. In the interests of dealing with these difficulties, tablet tracking by computer video was greatly hampered by their identical size and similar shape, keeping a far distance from the traditional applications of image recognition, such as face recognition or vehicle video monitoring. Nevertheless, light intensity reflective energy and ANN recognition have remarkable superiority in locating those scattered tablet objectives, which undoubtedly confirms its reliable results and high efficient performance. Based on this theoretical hypothesis, the labor intensities will be greatly reduced, and simultaneously the high precision of tracking processes can also be ensured.

According to the recently published literatures, it can be learned that image tracking or video tracking were generally employed in such conditions as moving vehicle monitoring or people tracking. Wang and Wang [1] have proposed an approach based on computer version and image processing technique for automatic grain tracking; through an efficient box-tracking-based method, presented by Fukuda et al. [2], mammalian cells (fibroblast) on microcarrier have been clearly recognized. At the same time, Dill et al. [3] studied the tracking cells supported by digital image processing and obtained a rather satisfactory result for multiobjective positioning during their high-speed movement. Kuba et al. [4] also made their contributions on automatic particle detection by one-class SVM from microscope image. Similar performances have been obtained by González et al. and so forth [5] on tracking olive trees in high-resolution satellite images.

On the other hand, Guarino et al. [6] have optimized the traditional image analysis approach. But their optimization process needs a much more computation storage and time interval than that of other traditional ones, which restricts its direct usages in real-time drug mass production; Marçal [7] presented new methods based on mathematical morphology and were suitable for grains of circular shape; Kim et al. [8] have successfully proposed a real-time approach for tracking the number of passing people by using one single camera. Similar research founding proposed by Professor Luque-Baena et al. can also be learned from [9, 10] as well. In the area of moving-objective image detection and its automatic classification, Wong et al. [11] used an example of outdoor people tracking for tourist-flow estimation in a constrained environment. According to the research result of Buczkowski et al. [12], the box-tracking method (BCM) shows a very high percentage of error in experimental practice, due to the identical shape and high-speed movement of those drug tablets to be studied. In order to cover this deficiency, Wang et al. [13] have proposed a new and efficient framework for pedestrian analysis and tracking, which consists of rule induction classifier and linear regression model. Agustin and Oh [14] preprocessed the detected foreground objects to eliminate pixel noise and small artifacts by performing opening morphology operation. Besides, Lien et al. [15] also paid high attention on a novel vehicle detection method without background modeling simultaneously. Zhan and Luo [16] made their contribution on system design of real-time vehicle recognition based on video for Windows (AVI) files; the development of a block-based real-time tracking system has been concluded by Park et al. [17] and Głowacz et al. [18] and Park et al. [19] have paid their attentions to the optical flow calculation and the area-based decision rule, respectively, which helps in acquiring reliable image tracking results. When discussing the cross-correlation in pattern recognition, Hong [20] used correlation coefficient of fuzzy numbers under arithmetic operations. Park et al. [21] have studied the correlation coefficient of interval-valued intuitionistic probability sets to multiple attribute group decisions. Ye [22] and Son [23], respectively, proposed probability decision-making method and fuzzy entropy determination for investigating the mutual-relationships among different factors. Other related research results can be learned from [24–26]. Furthermore, literature [27–29] also presented their latest progresses in cross-correlation mechanisms while such evaluation approaches as intuitionistic probability, interval-valued probability sets, and gamma rank correlation coefficient were, respectively, described in [30–32].

Since these commonly used approaches were found being influenced by traditional limitations, the real-time tablet tracking cannot be ensured easily; more importantly, due to the fact that most existing image tracking technologies focus on human tracking or vehicle monitoring, which can be distinguished easily by the different shapes and the widely divergent sizes of those targeted objects in practice [33–35], the high similarity objective tracking method suitable for high-speed drug mass production still remains unstudied and undeveloped, that becomes a research margin of advanced video inspection in these years.

This paper is structured as follows.  Section 1 outlines the importance and necessity of objective tablet tracking.  Section 2 describes the theoretical foundation of light intensity reflective energy surface.  Section 3 presents mathematical properties for describing the reflective energy surfaces of drug tablets, Section 4 discusses the detailed tracking processes, with tracking discussions and performance comparisons mentioned in Section 5, and finally Section 6 concludes this paper as respected.

## 2. The Theoretical Foundation of Light Intensity Reflective Energy Surface

**Figure 1:**
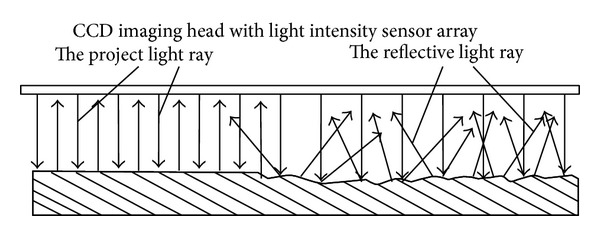
The light reflective effect caused by different surface topography areas, which composes an original property demonstration for calibrating the inflective-energy surface properties of drug tablets.

During the imaging process, the light was projected vertically thereafter a clear image can be obtained for showing the surface topography and geometrical shape of objective tablet. But, at the same time, the unsmooth surface topography on those drug tablets can also be identified due to their different light reflective effects.  Figure 1 shows the light reflective effect according to different topography areas, which composes an original property demonstration for calibrating the inflective-energy of objective tablet in this experiment. Since different topographic areas on objective tablet present different reflective effects of light, an average-distributed reflective energy condition of light intensity can be obtained on the smooth part of tablet surface. When the lighting ray casts on the objective surface to be observed, it will be reflected to CCD unit in the form of roughly parallel light routes; thus, an image with uniform distribution of light intensity will be gotten and a low concentration of lightness energy can also be shown. On the contrary, the light illumination on the rugged areas of tablet surface brings about the diffuse reflection of lighting rays, which results in the ununiformed concentration of lightness energy. Based on this theoretical presumption, the topography properties of objective drug tablet can be clearly classified with the reflective light intensity measurement, the computation of illumination energy, and the characteristic modeling of energy distribution.

## 3. Surface Mathematical Properties

Since the reflective energy of light intensity demonstrated by one given image pixel can be regarded as the height value in the *z* axis, thereafter the reflective energy distribution of the whole tablet image can be described in the form of free-form surface, from the perspective of spatial surface fitting domain. Based on this theoretical foundation the following physical properties have been proposed, for the sake of calibrating the established reflective energy surface of objective tablet in a quantitative way [36, 37].

**Property 1 .**

As it was well known that surface elasticity demonstrates the transformation resisting capability of one objective spatial surface shape under the force impact caused by external force loading, it becomes a typical index to calibrate the shape properties of spatial surfaces in a mathematical coordinate system, and simultaneously surface elasticity shows the concentricity level of characteristic distribution in the form of elasticity value as well. In this experiment, Property 1 demonstrates the elasticity distribution concentrating on one certain topography section, which makes it being conveniently used to describe topography properties from the perspective of elasticity variance distribution:

(1) φ=α1∑i=1mWui2+β1∑i=1mWuui2+α2∑j=1nWvj2+β2∑j=1nWvvj2 +α1α2β1β2∑i=1m ∑j=1nWuivj2−2f(u,v)W.

Here, *W* denotes an objective topography surface in the form of B-spline basis function; *W*_*u*_, *W*_*v*_, *W*_*uu*_, *W*_*vv*_, and *W*_*uv*_ are the partial derivatives of *W* in the first order, second order, and hybrid state of *u*, *v* axes, respectively; *α*_1_, *α*_2_, *β*_1_, and *β*_2_ are the given coefficients, *f*(*u*, *v*) denotes a given function of surface vector, and finally *m*, *n* denote the order amounts of surface vector in *u*, *v* axes.

**Property 2 .**

Energy of surface plays a prominent role as fairness function in the occasions of geometric modeling or surface microproperty analysis. Therefore, energy distribution can be computed and quantified to denote its belonged experimental conditions and geometric properties. This property defines the scattering level of surface energy in a mathematical sense, for a high value shows a more decentralized scattering of surface energy:

(2) ζ=∑i=1m∫ΩSu(ui)2du+∑i=1m ∑i=1m∬i∈ΩSuu(uii)2du du +∑j=1n∫ΩSv(vj)2dv+∑j=1n ∑j=1n∬j∈ΩSvv(vjj)2dv dv +∑i=1m ∑j=1n∬ΩSuv(uivj)2du dv.

Here, *S*_*u*_(*u*_*i*_), *S*_*uu*_(*u*_*ii*_), *S*_*v*_(*v*_*j*_), *S*_*vv*_(*v*_*jj*_), and *S*_*uv*_(*u*_*i*_ *v*_*j*_) denote the first order, second order, and hybrid derivatives of objective surface *f*(*u*, *v*) in *u*, *v* axes.

**Property 3 .**

Since surface construction highly depends on external loading effect, the fitted result will be deformed in an obvious scale. In the purpose of quantifying the influential effects caused by external loading, this property was newly proposed to calibrate the difference deviation and variation principle between the forced and original surfaces, as high amendment quantity demonstrates a relative critical deformation of resultant topography, with the definition shown as follows:

(3) ρ=−2∑i=muu∑j=mvvVi,j∯i,j∈ΩNi,su(u)Nj,sv(v)Ni,j(uv)×f(u,v)du dv.

Here, *N*_*i*,*s*_*u*__(*u*), *N*_*j*,*s*_*v*__(*v*), and *N*_*i*,*j*_(*uv*) denote the B-spline boundary control surfaces in *u*, *v* axes, respectively, with *V*_*i*,*j*_ denoting the transitional vector between two adjacent control vertexes impacted by external loading.

**Property 4 .**

The computation of the radial polynomial of one coordinate point (*x*, *y*, *z*) on free surface consists of three steps: computations of radial polynomials, radial basis functions, and radial polynomial moments, through projecting the coordinates of one control point on its belonged basis functions. *R*_*nm*_(*γ*) denotes the radial polynomial of one control point (*x*, *y*, *z*):

(4) $$\begin{array}{c} R_{nm}\left(\gamma \right)=\overset{(n-|m|)/2}{\underset{s=0}{\sum }}\frac{{\left(-1\right)}^{s}\left[\left(n-s\right)!\right]\gamma^{n-2s}}{s!\left(\left(n+\left|m\right|\right)/2-s\right)!\left(\left(n-\left|m\right|\right)/2-s\right)!}. \end{array}$$

Here, *n* denotes a positive integer or zero and *m* denotes an integer number and *n* − |*m* | = even number, with |*m* | ≤*n*; *r* denotes the vector length spaced from the origin point to one control point on surface *f*(*x*, *y*, *z*), defined as $\gamma =\sqrt{x^{2}+y^{2}+z^{2}}$, −1 < *x*, *y*,  *z* < 1.

**Property 5 .**

As the traditional Zernike moments have been extensively used, they receive much research attention in a number of fields: object recognition, terrain reconstruction, roughness segmentation, edge detection, and biomedical measurement. They have already become one of the most widely used families of orthogonal mathematical moments, owing to their extraordinary properties of being invariant or insensitive to any arbitrary rotation of the objective surface in the three-dimensional (3D) coordinate system. Based on the radial polynomial computation of one coordinate point (*x*, *y*, *z*) mentioned before, an improved Zernike moment was presented by focusing on their magnitude values as follows:

(5) $$\begin{array}{c} Z_{nm}=\frac{n+1}{\pi }\overset{1}{\underset{0}{\int }}\overset{2\pi }{\underset{0}{\int }}R_{nm}\left(\gamma \right)e^{jm\theta }f\left(\gamma ,\theta \right)\gamma \;d\gamma \;d\theta . \end{array}$$

**Property 6 .**

*P*(*u*, *v*) (0 < *u*, *v* < 1) was supposed to be the objective surface labeled by *m* control vertexes in *u* direction, with the node vectors being (*u*_0_, *u*_0_, *u*_0_, *u*_0_,…, *u*_*m*−4_,*u*_*m*−3_, *u*_*m*−3_, *u*_*m*−3_, *u*_*m*−3_), *u*_0_ = 0, *u*_*m*−3_ = 1, and *n* control vertexes in *v* direction, with the node vectors being (*v*_0_, *v*_0_, *v*_0_, *v*_0_,…, *v*_*n*−4_, *v*_*n*−3_, *v*_*n*−3_, *v*_*n*−3_, *v*_*n*−3_), *v*_0_ = 0, *v*_*n*−3_ = 1. Two signless integral numbers *n*_1_, *n*_2_ were employed for *m* ≤ 2^*n*1^ + 3, *n* ≤ 2^*n*2^ + 3, and *V* represents the congregation of (*m* − 2)(*n* − 2)/2 parametric points in *P*(*u*, *v*): (*u*_*k*_, *v*_*l*_) (*k* = 0,1,…, *m* − 3; *l* = 0,1,…, *n* − 3). Then, the fairing error of objective surface was defined as

(6) $$\begin{array}{c} \varepsilon_{n_{1},n_{2}}=\frac{\max_{(u,v)\in V}\left|P\left(u,v\right)-P_{n_{1},n_{2}}\left(u,v\right)\right|}{\left|P\left(u,v\right)\right|}\times 100\%;\\ \bar{S_{n_{1},n_{2}}}=1-\frac{\varepsilon -\varepsilon_{n_{1},n_{2}}}{\varepsilon_{n_{1}-1,n_{2}-1}-\varepsilon_{n_{1},n_{2}}}. \end{array}$$

Here, *P*_*n*_1_,*n*_2__(*u*, *v*) denotes the approximated surface of *P*(*u*, *v*) with *n*_1_ × *n*_2_ control vertexes, which can be obtained from the frequency decomposition of *P*(*u*, *v*) in its 3D coordinate lattice.

## 4. Experiment for Drug Tablet Tracking

### 4.1. The Arrangement of Experimental Device

**Figure 2:**
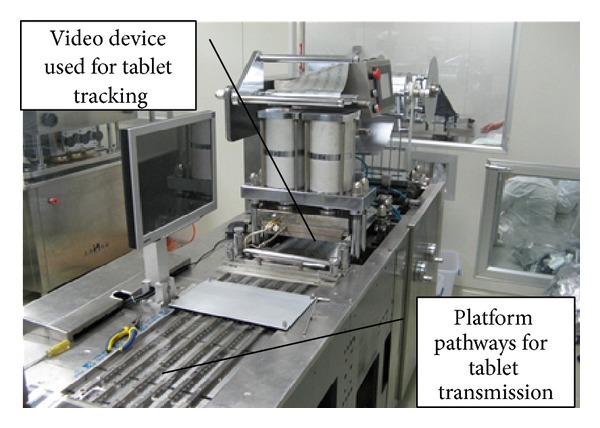
The video inspection platform employed in this experiment for tracking drug tablets.

Stainless steel material was employed to manufacture the experimental platform, with the purpose of avoiding the high light reflective index and mirror effect of background material, causing these two physical phenomena to reduce the contrast ratio and blur the objective clearance in video image remarkably [38, 39]. On the stainless steel pathways, the drug tablets were scattered into the monitoring field for high-speed video detection.  Figure 2 illustrates the video inspection platform employed, including the video device for tablet tracing and the platform pathways for tablet transmission. For the convenience of tracing these tablet objectives with high accuracy and efficiency, we subdivided the whole imaging field into several blocks by the size of 100 mm × 100 mm and then used the DMIRM CCD high-speed imaging system manufactured by German LEICA to capture the moving processes of drug tablets at the rate of 24 frames per second (FPS). It is noteworthy that this video imaging system consists of three essential components: a precise high-speed CCD head equipped with light intensity sensor array, a PC equipped with video processing board, and a set of multidimensional triangulation processing software for ensuring high clearances of video image frames. Since the light source being used is a visible coaxial LED (220 V; 20 W; 700 nm) combined with diffraction grating that generates a fixed number of stripes, which enables the system to capture more than 450,000 pixels in one single image frame. The range measurements were computed by using triangulation operation between the binary digital images and the calibrated imaging plane locations. The high-speed CCD microscopic camera was able to capture image pixel data in 0.01 ms (enabling image acquisition from a continuously moving manipulator) and process data array in 10 ms and has a measurement deviation of 1/20000 in the field of view. This CCD imaging head has been attached to a* DMIRM* 3-axis directly driven configuration manipulator for self-adaptively video imaging; thus, it eliminates image distortion caused by inhomogeneous light and keeps a rapid reaction and stable tracing performance simultaneously. For the purpose of improving the detection precision or dynamic tracing of drug tablets to the precise scale (1~5 mm), we predetermined the imaging depth as 0.3 m; therefore, the planar platform can be focused. On the other hand, the position overlap of drug tablets can be effectively avoided due to the continual high-frequency vibration of the experimental platform pathways, which reduces the probability of erroneous judgment to the maximum extent. In this experiment, the exposure time for digital imaging was supposed to be 0.5 s whereas the moving speed of drug tablet is about 3.5 m/s; therefore, one given tablet moves 1.75 m during the whole imaging process. In order to reduce the blur effect on the captured images, image sharpening was used to keep the high resolution of those studied objective tablets and thus accurate pattern recognition can be ensured.

### 4.2. The Computation of Transitional Vectors between Surface Control Points

In order to establish surface model for describing the light intensity reflective energy of drug tablets, the obtained energy value at the specific coordinate position of one pixel can be demonstrated as the *z*-axis value in the vertical direction; therefore, it was considered as the spatial coordinate of one control point. Under usual illumination, the external surface of drug tablet presents a constant situation of light intensity and uniform distribution of grey level. Based on this precondition, the three-dimensional (3D) transitional vectors can be computed.

During the capturing of digital images, the observer sight line keeps orthogonal intersecting with the imaging plane called *XOY*. The light intensity of one surface point (*x*, *y*) can be denoted by *I*(*x*, *y*), its project vector was determined as [*pi*, *qi*, −1], and then the normal vector of its reflective energy surface can be described as [*p*, *q*, −1]. In this paper, *θ* denotes the included angle that lies between the project vector of lighting and the normal vector of reflective energy surface. The reflective intensity of lighting energy in the direction of normal vector can be computed as [40, 41]

(7) $$\begin{array}{c} E\left(x,y\right)=I\left(x,y\right)\rho \cos\theta . \end{array}$$

Here, *E*(*x*, *y*) denotes the reflective energy intensity of the image pixel (*x*, *y*), whereas *I*(*x*, *y*) denotes the incident illumination intensity and is supposed as a constant number; *ρ* denotes the light reflectivity level of objective tablet surface (constant number).

Then, the cosine value of this included angle *θ* can be expressed as [42]

(8) $$\begin{array}{c} \cos\theta =\frac{pp_{i}+qq_{i}+rr_{i}}{\begin{array}{cc} \sqrt{p^{2}+q^{2}+r^{2}} & \sqrt{p_{i}^{2}+q_{i}^{2}+r_{i}^{2}} \end{array}}. \end{array}$$

The energy intensity of one image pixel (or called as the reflective light intensity at the objective pixel) can be identified as

(9) $$\begin{array}{c} E\left(x,y\right)=I\left(x,y\right)\rho \frac{pp_{i}+qq_{i}+rr_{i}}{\begin{array}{cc} \sqrt{p^{2}+q^{2}+r^{2}} & \sqrt{p_{i}^{2}+q_{i}^{2}+r_{i}^{2}} \end{array}}. \end{array}$$

**Figure 3:**
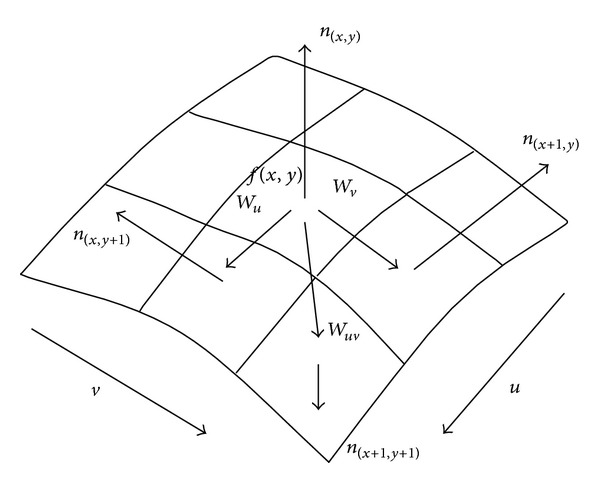
The transitional vectors and their spatial distribution on the fitted reflective energy surface.

Figure 3 shows the transitional vectors between surface control points and their spatial distribution. Through presenting a specific image pixel at (*x*, *y*), this research obtained the normal vector of energy surface: *n*_(*x*,*y*)_ = [*p*_1_, *q*_1_, *r*_1_], together with other normal ones at its neighboring: *n*_(*x*+1,*y*)_ = [*p*_2_, *q*_2_, *r*_2_], *n*_(*x*,*y*+1)_ = [*p*_3_, *q*_3_, *r*_3_], and *n*_(*x*+1,*y*+1)_ = [*p*_4_, *q*_4_, *r*_4_]. On this basis, the tangent vectors *W*_*u*_, *W*_*v*_, and *W*_*uv*_ at the positions of (*x* + 1, *y*), (*x*, *y* + 1), and (*x* + 1, *y* + 1) can be determined, respectively:

(10) Wu=n(x,y)×n(x+1,y)=|ijkp1q1r1p2q2r2|=[|q1r1q2r2|,|r1p1r2p2|,|p1q2p2q2|]=(u1,u2,u3),Wv=n(x,y)×n(x,y+1)=|ijkp1q1r1p3q3r3|=[|q1r1q3r3|,|r1p1r3p3|,|p1q2p3q3|]=(v1,v2,v3),Wuv=n(x+1,y)×n(x,y+1)=|ijkp2q2r2p3q3r3|=[|q2r2q3r3|,|r2p2r3p3|,|p2q2p3q3|]=(uv1,uv2,uv3).

The secondary partial derivative vectors were identified as *W*_*uu*_ = (*u*_11_, *u*_22_, *u*_33_) and *W*_*vv*_ = (*v*_11_, *v*_22_, *v*_33_), which demonstrates the variance ratio of those tangent vectors in *u* and *v* directions, respectively. Therefore, the finite difference method can be used to determine these two items:

(11) Wuu1=(uu1,uu2,uu3)(x,y)=Wu2−Wu1=(u1,u2,u3)(x+1,y)−(u1,u2,u3)(x,y)Wuu2=(uu1,uu2,uu3)(x+1,y)=Wu3−Wu2=(u1,u2,u3)(x+2,y)−(u1,u2,u3)(x+1,y)  ⋮Wuuk=(uu1,uu2,uu3)(x+k,y)=Wu(k+1)−Wuk=(u1,u2,u3)(x+k+1,y)−(u1,u2,u3)(x+k,y)Wvv1=(vv1,vv2,vv3)(x,y)=Wv2−Wv1=(v1,v2,v3)(x,y+1)−(v1,v2,v3)(x,y)Wvv2=(vv1,vv2,vv3)(x,y+1)=Wv3−Wv2=(v1,v2,v3)(x,y+2)−(v1,v2,v3)(x,y+1)  ⋮Wvvl=(vv1,vv2,vv3)(x,y+l)=Wv(l+1)−Wvl=(v1,v2,v3)(x,y+l+1)−(v1,v2,v3)(x,y+l).

Based on the above-mentioned process, the normal vector *n*_(*x*,*y*)_ = [*p*_1_, *q*_1_, *r*_1_] of one specific surface point can be determined the same as its partial derivative vectors at the 1st and 2nd order, respectively. Consider

(12) $$\begin{array}{c} W_{u}=\left(u_{1},u_{2},u_{3}\right);\;\;W_{uu}=\left(u_{11},u_{22},u_{33}\right);\\ W_{v}=\left(v_{1},v_{2},v_{3}\right);\;\;W_{vv}=\left(v_{11},v_{22},v_{33}\right);\\ W_{uv}=\left(uv_{1},uv_{2},uv_{3}\right). \end{array}$$

### 4.3. The Establishment of Light Intensity Reflective Energy Surface

**Figure 4:**
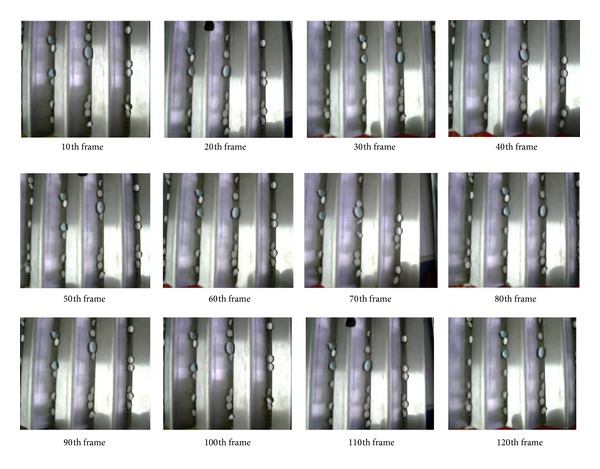
Typical image frames obtained from the tablet video for objective tracking in high similarity.

**Figure 5:**
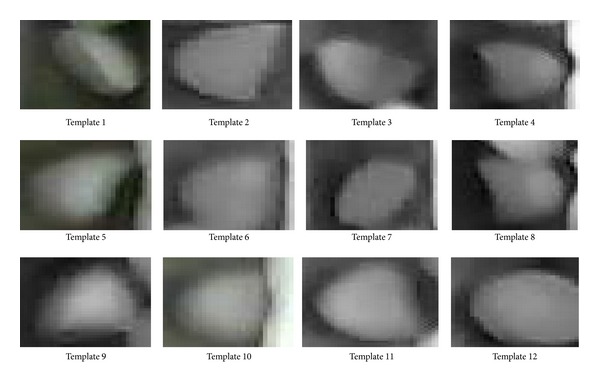
Objective tablet templates for image tracking in this experiment.

Figure 4 shows some typical image frames for objective tracking, the amount of drug tablets being seen in the monitoring area was strictly limited under 20 by predetermined setting, in the interest of keeping clear shape description and instantaneous tracking of those objective tablets. Through characteristic statistical analysis of drug tablets in geometric shapes, 12 tablet templates in typical geometric shapes and different laying angles were selected out for the following pattern recognition, with all possible shape morphologies and variant position arrangements during monitoring process being fully included, as Figure 5 demonstrates. Compared with other mathematical criterions, using tablet template facilitates the following determination of their geometrical characteristics in a clearer way. Since energy optimization fitting becomes a frequently used modeling method supported by mathematical programming and structure optimization, it regards the studied reflective energy surface providing the minimum physical distorting energy as its ultimate objective; therefore, various means of energy restriction and employed loadings were applied to achieve this goal in practice [43–45].

Energy optimization method regarding the laminose elastic deformation equation of elastic mechanics as its mathematical references:

(13) Esurface=∬[[α11Wu2+2α12WuWv+α22Wv2+β11Wuu2+2β12Wuv2+β22Wvv2]−2Wf(u,v)]du dv.

Here, *W* denotes the objective surface represented in *u*, *v* axes; *W*_*u*_, *W*_*v*_, *W*_*uu*_, and *W*_*vv*_ denote the first-order and second-order derivative vectors of *W* in *u* and *v* axes, respectively. *W*_*uv*_ denotes a mixing derivative vector, *α* and *β* were given parameters, and *f* denotes a given vector function to be approximated.

In practical modeling, *α* and *β* often denote the referential parameters of material characteristic; *f* denotes the external employed loading used for energy surface control. Under normal condition, the influence results exerted by *α*, *β*, and *f* will be generally neglected and thereafter valuated as 1, 1, and 0, respectively. Consider

(14) $$\begin{array}{c} E_{\text{surface}}=\iint \left[\left[\begin{array}{c} W_{u}^{2}+2W_{u}W_{v}+W_{v}^{2}\\ +W_{uu}^{2}+2W_{vv}^{2}+W_{vv}^{2} \end{array}\right]\right]du\;dv. \end{array}$$

By using the basis function of B-spline surface, the above-mentioned function was transferred into

(15) $$\begin{array}{c} w\left(u,v\right)=\sum_{\begin{array}{c} i=0,mu\\ j=0,mv \end{array}}V_{i,j}N_{i,su}\left(u\right)N_{j,sv}\left(v\right). \end{array}$$

Here, *V* denotes a control point of surface *w*(*u*, *v*); *mu* + 1, *mv* + 1 denote the number of control points in two axes; *su* and *sv* denote the power of studied surface; *N*_*i*,*su*_(*u*)*N*_*j*,*sv*_(*v*) are the B-spline basis function defined by *su*, *sv*, together with the knot vector denoted by *K*_*U*_, *K*_*V*_ (*U* = [*u*_0_, *u*_1_, *u*_2_,…, *u*_*m*+*s*+1_], *V* = [*v*_0_, *v*_1_, *v*_2_,…, *v*_*m*+*s*+1_]) as well. All variables in these equations were defined in *u* or *v* directions.

Equation (16) defines the first-order and second-order derivative vectors *w*_*u*_(*u*) and *w*_*uu*_(*u*):

(16) wu(u,v)=∑i=0,muj=0,mvVi,jNi,su′(u)Nj,sv(v);wv(u,v)=∑i=0,muj=0,mvVi,jNi,su(u)Nj,sv′(v);wuu(u,v)=∑i=0,muj=0,mvVi,jNi,su′′(u)Nj,sv(v);wvv(u,v)=∑i=0,muj=0,mvVi,jNi,su(u)Nj,sv′′(v);wuv(u,v)=∑i=0,muj=0,mvVi,jBi,su′(u)Bj,sv′(v).

Substituting (16) into (14) and letting *f* = 0, then

(17) $$\begin{array}{c} E=\overset{1}{\underset{0}{\iint }}\sum_{\begin{array}{c} i=0,mu\\ j=0,mv \end{array}}V_{i,j}\sum_{\begin{array}{c} k=0,mu\\ l=0,mv \end{array}}V_{k,l}*\left[\begin{array}{c} \alpha_{11}N_{i,su}^{\prime }\left(u\right)N_{j,sv}\left(v\right)N_{k,su}^{\prime }\left(u\right)N_{l,sv}\left(v\right)\\ +2\alpha_{12}N_{i,su}^{\prime }\left(u\right)N_{j,sv}\left(v\right)N_{k,su}^{\prime }\left(u\right)N_{l,sv}\left(v\right)\\ +\alpha_{22}N_{i,su}^{\prime }\left(u\right)N_{j,sv}\left(v\right)N_{k,su}^{\prime }\left(u\right)N_{l,sv}\left(v\right)\\ +\beta_{11}N_{i,su}^{\prime \prime }\left(u\right)N_{j,sv}\left(v\right)N_{k,su}^{\prime \prime }\left(u\right)N_{l,sv}\left(v\right)\\ +2\beta_{12}N_{i,su}^{\prime \prime }\left(u\right)N_{j,sv}\left(v\right)N_{k,su}^{\prime \prime }\left(u\right)N_{l,sv}\left(v\right)\\ +\beta_{22}N_{i,su}^{\prime \prime }\left(u\right)N_{j,sv}\left(v\right)N_{k,su}^{\prime \prime }\left(u\right)N_{l,sv}\left(v\right) \end{array}\right]du\;dv. \end{array}$$

Then,

(18) Si,j,k,l=∬01[α11Ni,su′(u)Nj,sv(v)Nk,su′(u)Nl,sv(v)+2α12Ni,su′(u)Nj,sv(v)Nk,su′(u)Nl,sv(v)+α22Ni,su′(u)Nj,sv(v)Nk,su′(u)Nl,sv(v)+β11Ni,su′′(u)Nj,sv(v)Nk,su′′(u)Nl,sv(v)+2β12Ni,su′′(u)Nj,sv(v)Nk,su′′(u)Nl,sv(v)+β22Ni,su′′(u)Nj,sv(v)Nk,su′′(u)Nl,sv(v)]du dv.

Thus, (17) will be simplified into

(19) $$\begin{array}{c} E=\sum_{\begin{array}{c} i=0,mu\\ j=0,mv \end{array}}V_{i,j}\sum_{\begin{array}{c} k=0,mu\\ l=0,mv \end{array}}V_{k,l}*S_{i,j,k,l}. \end{array}$$

**Figure 6:**
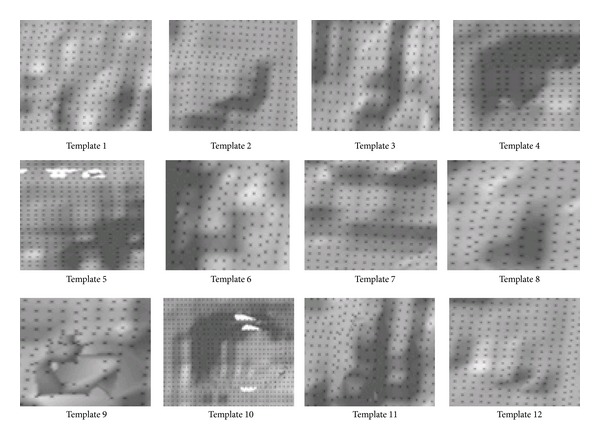
The established reflective energy surfaces of objective tablet templates.

Here, *S*_*i*,*j*,*k*,*l*_ denotes the integrating operation of one-known basis function; thus, the mathematical model of energy optimization surface can be transferred into the quadratic function of control vertexes *V* [46]. Based on this surface fitting method and the tablet templates shown in Figure 5, their corresponding reflective energy surfaces of light intensity can then be established with the resultant details being demonstrated in Figure 6.

### 4.4. The Application of ANN for Recognizing Surface Properties

In this study, back propagation (BP) learning algorithm, which has a unique learning principle, generally called delta rule, is used. As we all know, the back propagation learning algorithm is a common method for training artificial neural networks. Firstly, forward propagation of input vectors was realized to generate the propagation output activations, and then ANN uses the pattern target to generate the deviation of all output and hidden neurons. Based on this precondition, multiplying the calculation between the output deviation and the input activation was operated for obtaining the gradient vectors of those weigh values, which results into the percentage subtraction of their gradient vectors. Repeat these steps again until the performance of ANN network reaches a satisfied level. The three layers of the network architecture include the input layer, middle layer (hidden layer), and output layer. Layers include several processing units known as neurons. They connected with each other by variable weights to be determined. In this network, the input layer receives information from external source and passes this information to the network for data processing. The middle layer receives data from the input layer and does all information analysis. The output layer receives the processed information from the middle layer and sends the results to an external receptor [47].

A neuron in ANN network produces its input by processing the net input through nonlinear activation (transfer) function. The sigmoidal activation function is the most utilized one, which updates the weight and derivative values of ANN according to the resilient back propagation algorithm; therefore, it is usually trained for updating ANN networks. In this training algorithm, the update value for ANN weight is increased whenever the derivative value of its performance function has the same sign for two successive calculate iterations; on the other hand it is decreased according to the derivative value with respect to that weight changes sign from the previous iteration. If the derivative value is zero, then the update value for ANN weight remains the same. If the objective weight value continues to change in the same direction for several training iterations, then the magnitude of the weight change will be increased accordingly. In this experiment, we supposed that the input layer has* NI* neurons, with the middle layer and output layer having* NJ* and* NK *neurons, respectively, whereas the input elements for the *j*th neuron at the middle layer can then be described as

(20) $$\begin{array}{c} {\text{net}}_{j}=\overset{NI}{\underset{i=1}{\sum }}w_{ij}O_{i};\;j=1,2,\ldots ,NJ. \end{array}$$

Here, *w*_*ij*_ denotes the weight calibrating from the *i*th neuron on the input layer to the *j*th neuron on the middle layer, with *O*_*i*_ demonstrating the output result of the *i*th neuron on the input layer. Meanwhile, the resultant input of the *k*th neuron on the output layer can also be identified as

(21) $$\begin{array}{c} {\text{net}}_{k}=\overset{NJ}{\underset{j=1}{\sum }}w_{jk}O_{j};\;k=1,2,\ldots ,NK. \end{array}$$

Here, *w*_*jk*_ denotes the weight calibrating from the *j*th neuron on the middle layer to the *k*th neuron on the output layer, with *O*_*j*_ demonstrating the output result of the *k*th neuron on the middle layer. Through substituting the input neurons of the middle and output layer, as denoted by net_*j*_ and net_*k*_, into (20), the output results of these three layers can be illustrated as follows:

(22) $$\begin{array}{c} O_{i}={\text{net}}_{i}=x_{i},\\ O_{j}=f_{j}\left({\text{net}}_{j},\theta_{j}\right)=\frac{1}{1+e^{-({\text{net}}_{j}-\theta_{j})}},\\ O_{k}=f_{k}\left({\text{net}}_{k},\theta_{k}\right)=\frac{1}{1+e^{-({\text{net}}_{k}-\theta_{k})}}. \end{array}$$

Here, *θ*_*j*_ and *θ*_*k*_ denote the output threshold value used for assessing the computation results of the *j*th neuron on the middle layer and the *k*th neuron on the output layer, respectively. In this experiment, the gradient-based learning rule was used to train this established network, with the minimum mean square error (MSE) between the computation results caused by the network output and tablet template being predetermined as the ultimate train objective. The template amount for training was supposed to be *P*, and the input elements can then be denoted by *x*_1_, *x*_2_,…, *x*_*p*_, with the output elements being denoted by *y*_1_, *y*_2_,…, *y*_*p*_. Here, this experiment used *t*_1_, *t*_2_,…, *t*_*p*_ to denote the data vector consisted by training samples. Thus, MSE of the *p*th sample can be obtained as follows:

(23) $$\begin{array}{c} E^{p}=\frac{1}{2}\overset{NK}{\underset{k=1}{\sum }}{\left(t_{k}^{p}-y_{k}^{p}\right)}^{2}. \end{array}$$

Here, *t*_*k*_^*p*^ denotes the reference value of the *p*th sample for the *k*th output neutron, while *y*_*k*_^*p*^ denotes the practical output in the same condition. Therefore, the weight on the output layer can then be adjusted to

(24) $$\begin{array}{c} \Delta w_{jk}\left(n+1\right)=\eta \varepsilon_{k}^{p}O_{j}^{p}+\alpha \Delta w_{jk}\left(n\right);\\ \varepsilon_{k}^{p}=\left(t_{k}^{p}-y_{k}^{p}\right)f_{k}^{\prime }\left({\text{net}}_{k}^{p}\right). \end{array}$$

Here, *η* represents the learning rate, *α* denotes the momentum factor, and *δ*_*pk*_ denotes the deviation value between *t*_*k*_^*p*^ and *y*_*k*_^*p*^, which shows the difference between the reference value and the practical output of the *p*th sample caused by the *k*th output neutron [48]. Therefore, the weights of middle layer can then be adjusted into

(25) $$\begin{array}{c} \Delta w_{ij}\left(n+1\right)=\eta \varepsilon_{j}^{p}O_{i}^{p}+\alpha \Delta w_{ij}\left(n\right);\\ \varepsilon_{j}^{p}=f_{j}^{\prime }\left({\text{net}}_{j}^{p}\right)\overset{NK}{\underset{k=1}{\sum }}\delta_{pk}w_{jk}. \end{array}$$

When focusing on the ANN Testing process, it should be noted that the idea which we have used for testing neural networks and generally applying test vectors is that we use a multicase test vector to determine the decision strength of this neural network. For the final evaluation, we captured 100 images describing the random movement cases of drug tablets. Such a large testing case amount was used to minimize the uncertainty of the objective classifier's performance estimation in this experiment. Then, according to the reflective energy surfaces of those drug tablets, we input their corresponding property vectors to the established ANN and check the obtained output vectors in sequence. Based on the testing operations, this neural network reaches the desired performance; thus, a high improvement in the video detection of drug tablets can be observed.

**Figure 7:**
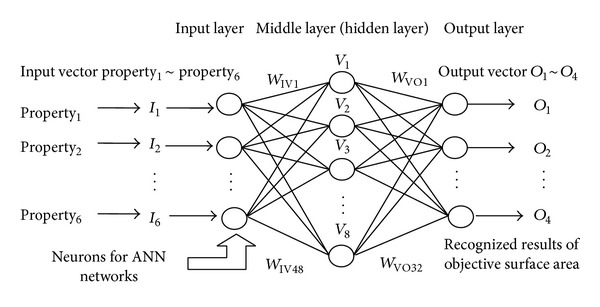
Schematic illustration of ANN used for surface property recognition and tablet tracking.

The weight values in ANN network markedly affect the classification of tablet templates, which keep a close correlation with the computation or tracking errors in the proposed experimental conditions; they were also easily impacted by the arrangement of neural neutrons and input/output vectors as well as weight values provide correlation among network layers, which ensures the feasibility of recognizing the geometrical shapes of tablet template and the property distribution of its reflective energy surface with the self-adaptive control of ANN. As Figure 7 shows, the detailed setup of ANN network is used, and it was observed that, for the purpose of realizing the improvements in network recognition and objective classification, the weights between input and middle layers should be kept in a relatively stable state, which provides a useful tool to markedly eliminate the external error-training interference caused by noisy sample vectors or error distribution gradient of input data. Simultaneously, the weights between middle and output layers should be kept in a radically distinction state, which ensures a clearer classification of tablet properties, especially when the self-adaptive learning rule was being employed. Based on these preconditions, ANN network was gradually optimized for improving its recognition performance [49, 50].

## 5. Tracking Process Discussions and Performance Comparisons

With the trained neural network and the surface properties computed by (1)–(6), they were used as the input vector for recognizing the tablet templates:

(26) Input=[I1,I2,I3,I4,I5,I6]=[property1,property2,property3,property4,property5,property6].

Then, the output vector can be predetermined as [*O*_1_, *O*_2_, *O*_3_, *O*_4_] by using forward propagation algorithm; the maximum possible reiteration times were determined as 15000, in order to prevent the case of training threshold does not be met; the node number of the hidden layer was 2, the learning rate coefficient was supposed to be 0.45, the momentum factor was supposed to be 0.065, the train step was supposed to be 0.15, and the interval illustration factor for recognition process was predetermined as 30. The error function was determined by (27), and it can also be employed to describe the ANN performance capability:

(27) $$\begin{array}{c} E_{\text{all}}=\overset{P}{\underset{p=1}{\sum }}E^{P}. \end{array}$$

This index examines if the prerequirement was satisfied or not: *E*_all_ < *ε*? If it was confirmed, then the training processes of ANN can be terminated or it will be iterated for the next time until this prerequirement is satisfied.

When discussing the determination of *ε*, through establishing the weights between network layers, then

(28) $$\begin{array}{c} \varepsilon =1-\xi \sqrt{\overset{12}{\underset{j=1}{\sum }}{\left|\Delta w_{kj}\left(1-\delta_{pk}\right)\right|}^{p}.} \end{array}$$

Here, *ξ* denotes a distinguishing coefficient located in [0,1]. Δ*w*_*kj*_ denotes the self-adaptive deviation value between the weight values calibrated from the *j*th neuron on the middle layer to the *k*th neuron on the output; *δ*_*pk*_ denotes the deviation value between *t*_*k*_^*p*^ and *y*_*k*_^*p*^. By using this threshold screening mechanism, the accuracy of pattern recognition can be ensured, and thereafter the overtraining phenomenon in ANN network could also be prevented. The greater this threshold value is, clearer the objective recognition result would be.

**Figure 8:**
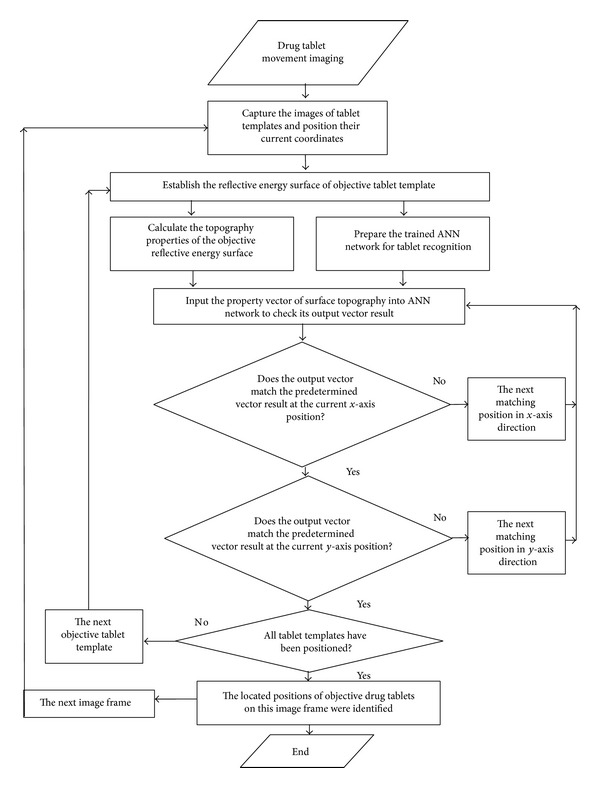
The program flow schematic of tablet template recognition and position justification.

**Figure 9:**
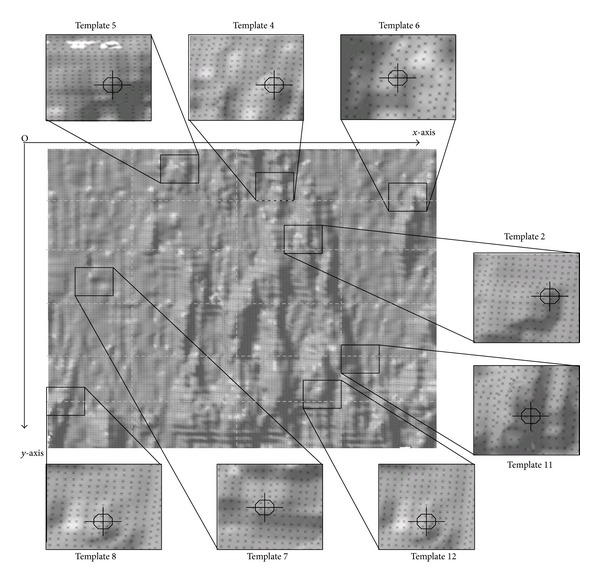
The position distributions of surface areas to best match their corresponding tablet templates on the 35th image frame.

**Figure 10:**
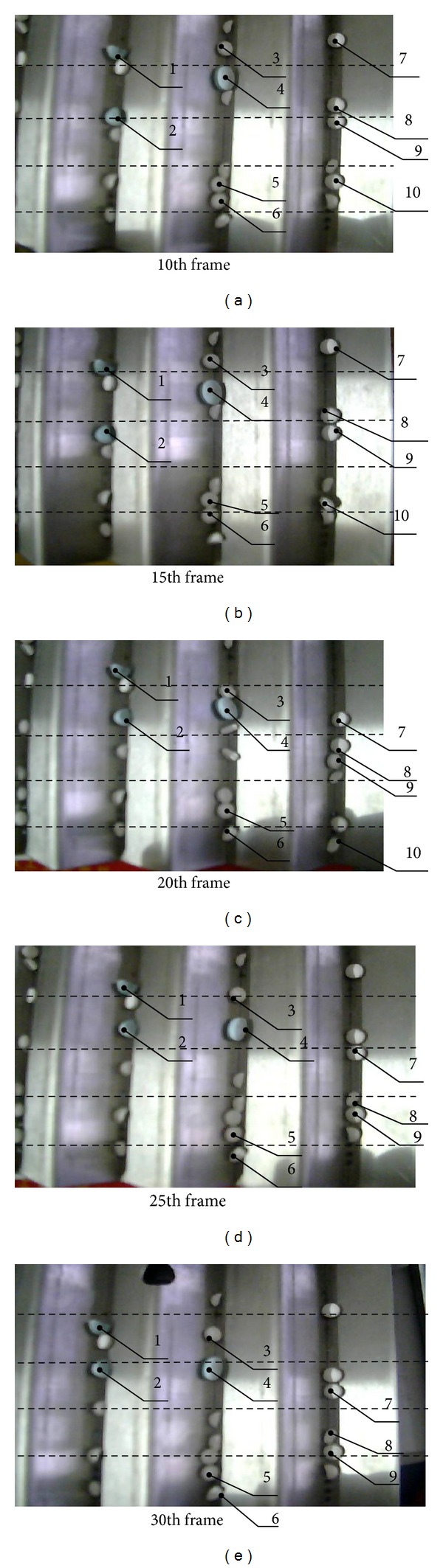
Representative video tracking results on different image frames, with the key objective tablets that have been labelled by numbers to show their moving processes in details, respectively.

Table 1 demonstrates the original and corrected weights of ANN. It is noteworthy that the original weights were tentatively selected with reference to (20)-(21), in the hope of reducing the computation deviation between the desired and practical output vectors to the minimum scale [51, 52].  Table 2 demonstrates the energy surface properties of tablet templates on the 35th image frame. In the interest of clearly describing the detailed process of tablet template recognition and its position justification, a program flow schematic was provided, with the details shown in Figure 8.  Table 3 describes the recognition results of surface properties; similar operations were repeatedly operated for times. Output vector composed by four digits is used to denote the specific objective template. For example, $\left[\begin{array}{cccc} 0 & 0 & 0 & 1 \end{array}\right]$ represents template 1, $\left[\begin{array}{cccc} 0 & 0 & 1 & 0 \end{array}\right]$ represents template 2, $\left[\begin{array}{cccc} 0 & 0 & 1 & 1 \end{array}\right]$ represents template 3, $\left[\begin{array}{cccc} 0 & 1 & 0 & 0 \end{array}\right]$ represents template 4,…, and $\left[\begin{array}{cccc} 1 & 1 & 0 & 0 \end{array}\right]$ represents template 12, with the mean values of recognition results based on repeatedly experiments being used. Through data comparison, the obtained results keep a close difference with those predetermined one, which undoubtedly confirms their effectiveness and accuracy. As one typical pattern recognition, Figure 9 shows the coordinate positions of surface areas best matching their corresponding tablet templates on the 35th image frame, with the black boxes showing the probable positions of the targeted tablets to be studied. In this figure, the most possible matching positions have been magnified and displayed in surrounding images for clearer description; the coordinate positions of template centers were highlighted by the focus marks. For better illustration of the tablet tracking result, 10 key tablets have been labeled with numbers, especially those close ones that were highly focused, as Figure 10 shows. We can observe that the practical operation with this algorithm can be smoothly identified, and those tablets in close position can also be distinguished clearly. Besides, as the interval distance between two adjacent dashed lines was predetermined as 30 mm, the instantaneous moving speeds of objective tablets can be calculated.

**Figure 11:**
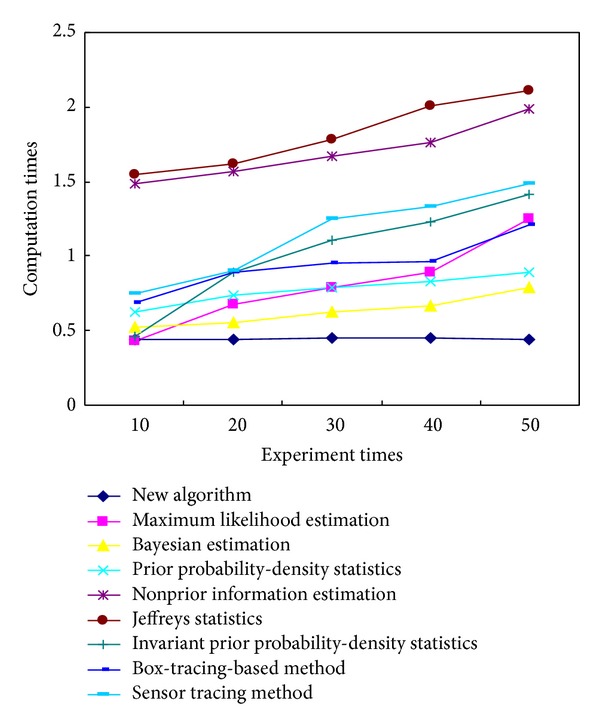
The variation tendency of average computation times for different experimental times with different objective tracking algorithms denoted by colored lines, the same as follows.

**Figure 12:**
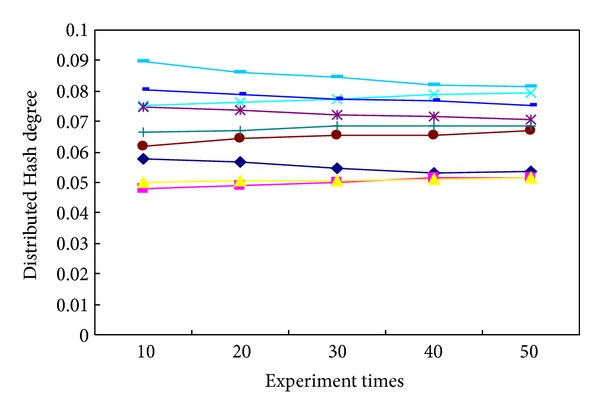
The variation tendency of average distributed Hash degrees for different experimental times.

**Figure 13:**
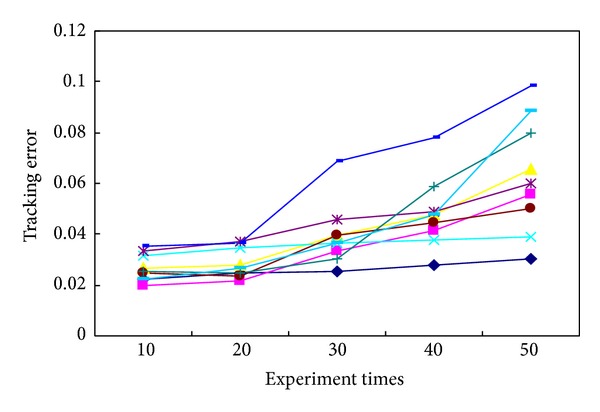
The variation tendency of average tracking errors for different experimental times.

**Figure 14:**
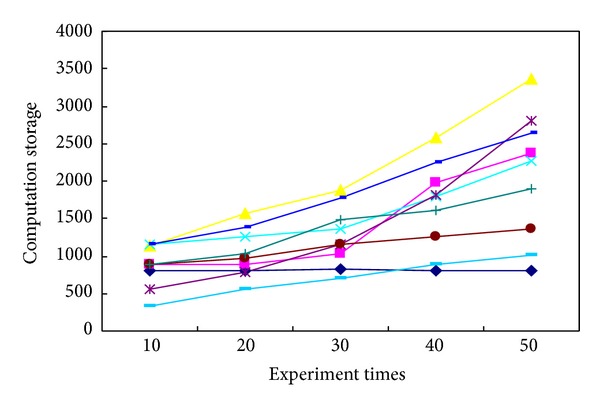
The variation tendency of average computation storages for different experimental times.

In order to prove the accuracy and validity of this newly proposed method, several commonly used tracking methods were investigated with reference to the published literatures, including the maximum likelihood estimation, the Bayesian estimation, the prior probability-density statistics, the nonprior information estimation, the invariant prior probability-density statistics, the Jeffreys statistics, the box-tracing-based method, and the sensor tracing method. Typical evaluation indexes were employed when an identical experimental condition has been considered: such as the computation time (the accurate time consumption in which the whole experiment uses the identical tracing platform; this research measures the computation time by means of the automated time counting instrument), the distributed Hash degree (the decentralized distributed complexity level that provides a lookup assessment index for algorithm evaluation similar to traditional Hash table, with a small value associated with the high-efficiently structure of tracing algorithm, and vice versa. This research calculates them through the performance process of one given algorithm and then makes a definite assessment on its performance capabilities as respected), the tracking error (the tracking ratio between the amount of error-tracking objectives to total ones, by which the tracing accuracy can be quantitatively identified. It can be calibrated by counting the amount of wrong-located tablets and total ones, resp., and then the ratio between them can be used as the tracking error), and the computation storage (the internal memory capacity used for the whole experimental computations; we calibrate them from the real-time performance indicator of the video tracking system that is being involved during the whole process of tablet tracking) [44–48]. After determining these evaluation indexes, their average performance capabilities were compared to each other, with the results shown in Table 4 after data equalization and value regularization. Since this experiment is repeatable, we take a detailed notes about the practical performances for 10, 20, 30, 40, and 50 times, and thereafter a statistical evaluation on their mean index values can be made by employing those mentioned algorithms, respectively. It can be seen from Figure 11 that, with the increment of experiment times, most of these tracking algorithms show a growth tendency in the average computation times. But it is noteworthy that a relative-stable performance capability can be ensured by using our newly proposed algorithm, which is kept at about 0.443 s throughout the whole process rather than changing radically. Similar performance evaluations can also be observed in the variation tendency schematics of Figure 12 with the average Hash degree (from 59.8% to 5.66%), the average tracking error (from 2.3% to 2.445%) in Figure 13, and the average computation storage (from 797.5 kb to 804.5 kb) in Figure 14.

From Table 4, it can be observed that the maximum likelihood estimation or the sensor tracing method has a relative superiority in the tracking error and distributed Hash degree; they can be widely used to investigate a stable statistical condition, by concerning the dynamic moving characteristics of identical tablets in huge cluster; the Bayesian estimation obtains a good performance result in the computation time; therefore, it will be more suitable to fast assess a simpler mutual relationship mechanism for the approximate shape properties of those drug tablets; the prior probability-density statistics method or the Jeffreys statistics approach have excellent computation performances when the tracking error or distributed Hash degree were highly emphasized, which ensure the accurate demonstrations of data relationship analysis and thereafter make a series of remarkable progresses when compared with other traditional methods; the nonprior information estimation method and the invariant prior probability-density statistics approach have extraordinary capabilities in the distributed Hash degree, which show a more precise algorithm complexity of data distribution in multidimensional statistical domain. Finally a good performance evaluation result can be obtained by using this newly proposed algorithm, especially in the computation time, computation storage, or distributed Hash degree. Performance comparison proves its validation and efficiency when similar shape or identical size of objective tablets should be taken into account, and the working estimation on how well these alternative methods perform on average can be facilitated as well.

## 6. Conclusions

A new image tracking approach for high similarity drug tablets based on light intensity reflective energy and ANN recognition has been investigated. Considering the practical condition and precision requirement, the reflective energy surfaces were established for modeling the high similarity geometric characteristics and surface topography of those targeted drug tablets, which results into the maximum deletion of external optical signal interferences or calibration error in practice, and simultaneously an accurate description of tablet objectives in the light intensity domain can also be ensured. Thereafter, ANN was used for recognizing those studied objective tablets with the computed surface properties, and then their instantaneous positions on one image frame can be identified clearly. Through repeating these steps on the sequential image frames, a series of tablet tracking results can be obtained. After being compared with other tracking methods in performance indexes and determined results, it can be learned that this newly proposed method solves numerous difficult problems characterized by monitoring precision, complicated calculations or tracking errors, and other signal interferences caused by mathematical calibration or pattern recognition. Therefore, the quantitative analysis of the objective tracking can be simplified, and the searching precision or computation efficiency can also be greatly improved, accordingly. Experimental computation and result analysis confirmed the validation and accuracy of this new method; new research ideas for real-time objective recognition or tablet tracking can be provided as well.

## Figures and Tables

**Table 1:** The original and corrected weight values of the established ANN in this experiment.

	The original weight value of ANN before training							
*W*_IV1_~*W*_IV8_	2.358	1.298	−3.114	5.558	−6.025	−0.553	3.339	6.554
*W*_IV9_~*W*_IV16_	−5.159	0.587	1.265	−3.558	−5.554	7.663	2.663	−2.226
*W*_IV17_~*W*_IV24_	0.668	−5.487	−6.587	9.558	3.214	3.271	1.098	−3.554
*W*_IV25_~*W*_IV32_	6.558	1.245	−6.584	−5.145	9.552	1.118	2.337	6.524
*W*_IV33_~*W*_IV40_	−3.224	−4.859	1.548	4.887	3.225	−3.876	8.098	−6.554
*W*_IV41_~*W*_IV48_	2.335	2.982	3.112	−3.442	7.078	1.987	9.003	2.338
*W*_VO1_~*W*_VO8_	3.265	2.554	−6.254	9.551	3.025	2.336	−8.476	−2.114
*W*_VO9_~*W*_VO16_	1.487	6.584	4.559	−6.002	5.554	4.098	−4.442	6.335
*W*_VO17_~*W*_VO24_	−6.954	8.885	3.654	8.201	−6.002	2.221	8.098	−6.598
*W*_VO25_~*W*_VO32_	6.547	−6.548	6.554	8.887	−6.254	−2.189	3.228	0.621
*W*_VO29_~*W*_VO32_	2.337	−3.072	4.976	−0.887	7.765	2.037	3.047	5.887

	The corrected weight value of ANN after training							

*W*_IV1_~*W*_IV8_	3.334	4.228	2.998	2.882	2.332	3.728	2.391	3.311
*W*_IV9_~*W*_IV16_	−0.887	6.447	−8.472	8.472	8.482	5.772	5.339	4.591
*W*_IV17_~*W*_IV24_	2.337	6.992	−1.993	6.472	3.442	6.102	5.281	5.993
*W*_IV25_~*W*_IV32_	5.823	9.073	4.378	3.448	5.312	9.082	−0.998	−2.921
*W*_IV33_~*W*_IV40_	9.008	2.774	7.461	5.729	7.422	5.698	−9.003	3.549
*W*_IV41_~*W*_IV48_	1.894	−8.372	8.472	5.773	9.082	4.332	−5.211	5.281
*W*_VO1_~*W*_VO8_	−8.774	0.987	4.228	8.728	9.228	6.937	7.381	−6.391
*W*_VO9_~*W*_VO16_	5.662	8.443	−0.879	8.227	−8.492	2.184	1.391	−5.112
*W*_VO17_~*W*_VO24_	8.447	9.622	2.398	2.173	7.472	−7.471	4.291	5.295
*W*_VO25_~*W*_VO32_	0.182	8.446	1.094	7.422	4.442	4.552	5.281	−3.591
*W*_VO29_~*W*_VO32_	−4.372	6.364	4.553	−0.921	7.311	6.082	8.391	−8.422

**Table 2:** The reflective energy surface properties of representative templates on the 35th image frame, which were employed as the input vector of ANN for pattern recognition, the same as the other surface property groups.

	The input values of the surface properties					
	*Property 1*	*Property 2*	*Property 3*	*Property 4*	*Property 5*	*Property 6*
1	3587.23	548.52	1587.9	10155.4	956.6	0.2669
2	1035.69	669.52	2036.5	12336.5	875.2	0.3022
3	2598.32	716.55	3544.5	10269.5	665.2	0.3654
4	4782.61	215.84	3055.8	9854.6	365.4	0.1485
5	5548.32	985.36	6248.2	9965.5	302.1	0.3954
6	3699.14	888.36	5598.7	7825.5	954.6	0.3022
7	6685.26	441.56	6694.8	6359.4	865.7	0.2035
8	1334.69	332.58	6025.8	9806.5	1012.5	0.3144
9	4026.54	698.22	4785.9	11025.4	1124.5	0.6022
10	4026.57	725.36	6698.5	12004.5	1325.9	0.3598
11	9833.21	889.65	4755.2	7985.6	968.8	0.4752
12	5476.32	102.54	6321.5	9586.6	1024.5	0.3301

**Table 3:** Representative recognition results of reflective energy surface properties for tracking those tablet templates on the 35th image frame.

Objective tablets	The recognized results (the obtained number vectors were the mean values based on 10 times of repeated experiments)		Recognition ratio
Template 1	$\left[\begin{array}{cccc} 0.0053 & 0.0024 & 0.0066 & 1.0035 \end{array}\right]$	Practical result vector	95.6%
	$\left[\begin{array}{cccc} 0 & 0 & 0 & 1 \end{array}\right]$	Predetermined vector	

Template 2	$\left[\begin{array}{cccc} 0.0014 & 0.0012 & 9.9971 & 0.0032 \end{array}\right]$	Practical result vector	94.6%
	$\left[\begin{array}{cccc} 0 & 0 & 1 & 0 \end{array}\right]$	Predetermined vector	

Template 3	$\left[\begin{array}{cccc} 0.0032 & -0.0011 & 1.0036 & 1.0042 \end{array}\right]$	Practical result vector	95.2%
	$\left[\begin{array}{cccc} 0 & 0 & 1 & 1 \end{array}\right]$	Predetermined vector	

Template 4	$\left[\begin{array}{cccc} -0.0018 & 1.0051 & 0.0041 & 0.0017 \end{array}\right]$	Practical result vector	92.9%
	$\left[\begin{array}{cccc} 0 & 1 & 0 & 0 \end{array}\right]$	Predetermined vector	

Template 5	$\left[\begin{array}{cccc} -0.0017 & 9.9981 & -0.0056 & 1.0088 \end{array}\right]$	Practical result vector	93.3%
	$\left[\begin{array}{cccc} 0 & 1 & 0 & 1 \end{array}\right]$	Predetermined vector	

Template 6	$\left[\begin{array}{cccc} 0.0055 & 1.0006 & 9.9968 & 0.0016 \end{array}\right]$	Practical result vector	95.1%
	$\left[\begin{array}{cccc} 0 & 1 & 1 & 0 \end{array}\right]$	Predetermined vector	

Template 7	$\left[\begin{array}{cccc} -0.0055 & 1.0041 & 0.9988 & 1.0044 \end{array}\right]$	Practical result vector	98.2%
	$\left[\begin{array}{cccc} 0 & 1 & 1 & 1 \end{array}\right]$	Predetermined vector	

Template 8	$\left[\begin{array}{cccc} 1.0058 & 0.0066 & 0.0014 & 0.0058 \end{array}\right]$	Practical result vector	93.9%
	$\left[\begin{array}{cccc} 1 & 0 & 0 & 0 \end{array}\right]$	Predetermined vector	

Template 9	$\left[\begin{array}{cccc} 1.0055 & 0.0041 & 0.0088 & 0.9944 \end{array}\right]$	Practical result vector	95.6%
	$\left[\begin{array}{cccc} 1 & 0 & 0 & 1 \end{array}\right]$	Predetermined vector	

Template 10	$\left[\begin{array}{cccc} 1.0043 & 0.0022 & 0.9997 & -0.0035 \end{array}\right]$	Practical result vector	93.9%
	$\left[\begin{array}{cccc} 1 & 0 & 1 & 0 \end{array}\right]$	Predetermined vector	

Template 11	$\left[\begin{array}{cccc} 1.0013 & 0.0025 & 0.9927 & -0.0089 \end{array}\right]$	Practical result vector	94.4%
	$\left[\begin{array}{cccc} 1 & 0 & 1 & 1 \end{array}\right]$	Predetermined vector	

Template 12	$\left[\begin{array}{cccc} 1.0036 & 1.0022 & 0.0088 & -0.0071 \end{array}\right]$	Practical result vector	97.5%
	$\left[\begin{array}{cccc} 1 & 1 & 0 & 0 \end{array}\right]$	Predetermined vector	

**Table 4:** Performance comparisons during drug tablet tracing by using different methods.

Method	Average computation time	Average distributed Hash degree	Average tracking error	Average computation storage
New algorithm	0.443 s	5.66%	2.445%	797.5 kb
Maximum likelihood estimation	0.678 s	4.90%	2.147%	883.1 kb
Bayesian estimation	0.558 s	5.03%	2.798%	1566.6 kb
Prior probability-density statistics	0.734 s	7.65%	3.446%	1251.4 kb
Nonprior information estimation	1.565 s	7.35%	3.709%	785.2 kb
Jeffreys statistics	1.623 s	6.44%	2.366%	975.1 kb
Invariant prior probability-density statistics	0.889 s	6.72%	2.477%	1032.5 kb
Box-tracing-based method	0.894 s	7.91%	3.674%	1377.3 kb
Senor tracing method	0.905 s	8.60%	2.668%	558.5 kb
